# Leveraging Large Language Models to Identify Engagement-Driving Features in Vaping-Related TikTok Videos: Cross-Sectional Study

**DOI:** 10.2196/76265

**Published:** 2025-11-20

**Authors:** Zidian Xie, Nanda Kishore Korrapolu, Amisha Dubey, Luchuan Song, Chenliang Xu, Karen M Wilson, AnaPaula Cupertino, Dongmei Li

**Affiliations:** 1 Clinical and Translational Science Institute, University of Rochester, 265 Crittenden Boulevard CU 420708, Rochester, NY, 14642-0708, United States, 1 5852767285; 2 Department of Computer Science, University of Rochester, Rochester, NY, United States; 3 Goergen Institute for Data Science and Artificial Intelligence, University of Rochester, Rochester, NY, United States; 4 Department of Pediatrics, University of Rochester, Rochester, NY, United States; 5 Department of Surgery, University of Rochester, Rochester, NY, United States

**Keywords:** electronic cigarettes, TikTok, artificial intelligence, AI, user engagement, e-cigarette, vaping, prevention, social media

## Abstract

**Background:**

Electronic cigarette (e-cigarette) use is prevalent in youth and young adults in the United States. TikTok (ByteDance), a popular social media platform among youth and young adults, has become a key avenue for disseminating e-cigarette-related videos, with promotional videos constituting the predominant form.

**Objective:**

This study aimed to identify key e-cigarette-related TikTok video features associated with high user engagement to assist with future video design for vaping prevention campaigns.

**Methods:**

We collected 1487 e-cigarette-related TikTok videos and related metadata posted between January 2023 and January 2024 using the TikTok API (application programming interface). We applied large language models GPT-4 and Video-LLaMA to extract video features (eg, promotion content, background, perceived sex, lifestyle, talking, cartoon, vaping tricks, and containing emojis) from e-cigarette-related TikTok videos. We randomly selected and hand-coded 25 videos to check the accuracy of 2 models in identifying these video features. We used a linear mixed effects model with random intercept to identify significant video features associated with high TikTok user engagement ([likes+shares+comments]/views).

**Results:**

Compared to the Video-LLaMA model, the GPT-4 model exhibited higher accuracy (83%‐100% vs 24%‐88%) in video feature identification. Notably, video backgrounds in cars (rate ratio [RR]=3.91, 95% CI 1.25‐12.20; *P*=.009) demonstrated significantly higher user engagement than in public spaces. Moreover, videos featuring young adults (RR=1.24, 95% CI 1.00‐1.53; *P*=.048), talking (RR=1.63, 95% CI 1.30‐2.05; *P*<.001), containing emojis (RR=1.88, 95% CI 1.48‐2.38; *P*<.001), or funny and silly content (RR=1.61, 95% CI 1.29‐2.00; *P*<.001) exhibited heightened user engagement. Conversely, videos with promotional content (RR=0.40, 95% CI 0.45‐0.81; *P*=.001) experienced lower engagement.

**Conclusions:**

TikTok video features like background settings, young adult presence, talking, and containing emojis and funny or silly content substantially enhance user engagement. These insights offer valuable guidance for designing compelling videos in vaping prevention campaigns to improve social media user engagement.

## Introduction

Electronic cigarette (e-cigarette) use has been prevalent among youth and young adults in recent years. Using National Youth Tobacco Survey data (2013‐2022), the cross-sectional analysis found that the prevalence of current e-cigarette use among US youth rose from 3.10% in 2013 to a peak of 20.18% in 2019, then declined and remained relatively stable in 2021 and 2022 (7.50% and 9.44%, respectively) [1]. Although the prevalence of e-cigarette use among middle school and high school students has decreased from 7.7% in 2023 to 5.9% in 2024, about 1.63 million youth still reported e-cigarette use in 2024 [2]. Results from the 2020 National Youth Tobacco Survey (NYTS) data analyses showed that the e-cigarette product characteristics (eg, e-cigarette flavors, concealability, and vape tricks), family and peer influence (family or friend use), curiosity, and replacing cigarettes were the main reasons for e-cigarette use [3]. While e-cigarettes are often cited for their potential as smoking-cessation aids [4], their long-term health effects remain uncertain [4,5], and therefore, there is broad consensus that youth access should be prevented [6,7]. Chemical analyses of e-cigarette aerosol have detected carcinogens like formaldehyde, acetaldehyde, acrolein, diacetyl, and toxic, carcinogenic metals such as chromium, nickel, and lead [8-12]. Human studies have shown that e-cigarette use was associated with several respiratory disorders (such as wheezing, asthma, and chronic obstructive pulmonary disease) [13-15] and mental health problems (such as depression) [16,17]. E-cigarette use costs the United States approximately US $15 billion annually in 2018 due to the US $2024 excess health care expenditures per e-cigarette user per year compared to never tobacco product users [18].

With over 1 billion monthly active users, TikTok is one of the most popular social media platforms among youth and young adults in the United States [19]. TikTok’s short video format, engaging content, and personalized recommendation system have attracted millions of youths and young adult users. The TikTok platform allows users to create, view, and share videos with posts leading to likes, shares, comments, and followers [20]. Recognizing the popularity of social media, the vaping industry and vaping proponents have prioritized social media to promote vaping. E-cigarette companies and vape shops promote e-cigarettes on TikTok by posting professionally designed videos using popular hashtags [21], creating fake user accounts to disseminate spam and favorable views [21,22], and providing celebrity sponsorship [23]. Although TikTok’s Community Guidelines prohibit content that facilitates the purchase, sale, trade, or solicitation of e-cigarettes, e-cigarette promotional posts remain prevalent on the platform [23].

In contrast to vaping promotion messages on social media, the number of social media posts educating or warning the public about the adverse health effects of vaping is minimal on all social media platforms [24,25]. To reduce the prevalence of vaping among youth, in September 2018, the US Food and Drug Administration (FDA) launched a vaping prevention campaign – “The Real Cost” on social media and other platforms to educate youth about the adverse health effects and risks of vaping [26]. However, the impact of the FDA’s “The Real Cost” vaping prevention campaign on Instagram (Meta) is limited partially due to low engagement [27]. For example, the FDA-sponsored TheRealCost account has a much lower median number of likes per Instagram post than the vaping promotion accounts [27]. Therefore, it becomes essential to understand and (especially) design vaping prevention messages with high user engagement for effective health communication in the community.

High user engagement, such as a high number of likes, might significantly impact user behavior [28]. Improving social media user engagement of vaping prevention messages can prevent vaping initiation and motivate cessation [29]. Vaping promotion posts have high engagement due to well-designed content and popular hashtags [21,22]. Some features (such as hashtags) in e-cigarette promotion posts with high user engagement could be integrated into the vaping prevention messages to increase user engagement. In addition, identifying the features of vaping promotion social media messages targeting vulnerable populations (such as youth) will facilitate future regulation of such social media posts. Thus, our study will include both vaping promotion and prevention posts. Current social media studies on e-cigarette-related TikTok videos focus on characterizing content rather than identifying high user engagement post features [30,31]. More recently, several studies have started to describe the features of social media posts associated with high user engagement [32,33]. Yet, none of the previous studies identified video features associated with high user engagement on TikTok. Video is popular on social media platforms and is more attractive than images and texts due to its combination of visuals, sounds, and motions to create an emotional connection with viewers through a storytelling mode, including facial expressions and different tones of voice [34]. Thus, identifying video features associated with high user engagement is important to attract TikTok users’ attention to vaping prevention and cessation content. This study aims to fill this knowledge gap by identifying video features from e-cigarette-related TikTok videos associated with high user engagement. Findings from this study are valuable in designing effective vaping prevention video messages for tobacco control and vaping cessation interventions.

## Methods

### Study Design

Our study is a cross-sectional TikTok post content analysis to identify important features associated with high user engagement in TikTok posts. All data collected and analyzed in our study are publicly available from the TikTok platform, and all results were reported in aggregate form to ensure user anonymity.

### Ethical Considerations

Our study has been reviewed by the University of Rochester Research Subjects Review Board (RSRB) office and was determined to meet federal and university criteria for exemption under Exempt Category 4 (Study ID: STUDY00009334). The RSRB determined that this study qualifies as secondary research involving existing data and, therefore, does not require informed consent. All publicly available TikTok videos included in the analysis were deidentified before use, and no compensation was provided to the TikTok users. Our study report followed the Strengthening and Reporting of Observational Studies in Epidemiology (STROBE) reporting guidelines [35].

### Data Collection

TikTok short videos (less than one minute) in English related to e-cigarettes from the United States were searched and downloaded using the TikTok research application programming interface (API) using keywords related to e-cigarettes, including “e-cig,” “vaping,” “e-cigarettes,” “e-cigs,” “ecig,” “ecigs,” “electroniccigarette,” “ecigarette,” “ecigarettes,” “vape,” “vapers,” “vapes,” “e-liquid,” “ejuice,” “eliquid,” “e-juice,” “vapercon,” “vapeon,” “vapefam,” “vapenation,” “juul.” [36]. The TikTok API has a limitation of around 100 on the number of videos we can collect each month. In February 2024, through the TikTok API, a total of 1487 TikTok videos (from 1064 unique creators) related to e-cigarettes posted between January 2023 and January 2024 were collected and confirmed manually in this study.

### Video Feature Extraction

The TikTok video feature list was derived primarily from our previous work—namely, our characterization of Instagram image features for e-cigarette content and our manual coding of e-cigarette videos on TikTok and YouTube [31-33,37,38]. We then reviewed sample TikTok videos to identify additional features that could affect engagement and, as a team, agreed on the final set of features for this study. The TikTok video features analyzed in this study included promotional content (including advertisements or promoting the e-cigarette use), celebrity endorsements, background setting, perceived sex (male or female, based on the observation), social events, young adult themes, lifestyle portrayals, e-cigarette devices (the presence of physical devices), smoking or vaping behaviors (the action of smoking or vaping regardless of the presence of physical e-cigarette devices), talking, singing, dancing, humorous or silly content, cartoons or animations, vaping tricks, and the use of emojis. We considered 2 cutting-edge large language models to extract video features from TikTok videos: GPT-4 [39] and Video Large Language Model Meta AI (Video-LLaMA-7B) [40]. Both models can understand the visual and auditory content in the video. Based on a reported AI labeling accuracy of 94% [41] and a prespecified 10% margin of error for the 95% CI, the single-proportion sample-size calculation indicated that 22 videos are required for our pilot evaluation of GPT-4 and Video-LLaMA-7B feature labeling. To examine which large language model performs better, we randomly selected 25 videos from the 1487 videos and manually labeled the proposed features by 2 human coders. Two human coders worked together side-by-side to watch these videos and label the defined video features. GPT-4 uses vision models to break down the visual component of a video frame by frame and detect objects, faces, text in the image, and other visual elements to help us identify features from the TikTok videos. In this study, to reduce data size and the demand for computational resources while still capturing meaningful information in the video, we adopted a frame sampling strategy, one image frame for every 15 frames, to represent the video [42]. In this study, the OpenAI API was used to interact with the GPT-4 model, and well-designed prompts were used to generate the desired and optimal output from the model. Table S1 in Multimedia Appendix 1 lists prompts used to automatically extract video features from the remaining 1462 TikTok videos using the GPT-4 API.

We used the GPT-4 API to categorize each video’s background into one of the following 6 groups based on the description of the background, including car, public space (club, studio, indoor and outdoor, medical building, restaurant, spacecraft, and other), private space (bathroom, bedroom, home, kitchen, room, and office), outside (beach, forest, garden, natural, space, campus, cave, urban, and natural), shop (shop and vape shop), and not detectable. Based on the description of the lifestyles from GPT-4, we classified the lifestyles into casual, leisure (fitness, gaming, and leisure), and no lifestyle (educational, health, industrial, vaping, working, or others) categories.

### Statistical Analysis

A linear mixed effects model with random intercept to account for multiple videos posted by the same user was used to model the association of TikTok user engagement with extracted features. We calculated the Spearman correlations among likes, shares, comments, and views of TikTok videos and found significant moderate-to-strong correlations across these metrics, as expected (Figure S1 in Multimedia Appendix 1). Given this interdependence, a more appropriate approach is to use overall engagement as the outcome variable, combining likes, shares, comments, and views using the formula applied by TikTok. The TikTok user engagement measure was calculated based on the formula [(number of likes+number of comments+number of shares)/(number of views+0.5)] X 100 [43]. For each video, add up all user actions (likes, comments, and shares) and then divide that total by the number of views. This gives interactions per view, so it reflects how actively viewers responded to the content, independent of how many people saw it. The value of 0.5 is added to the formula to minimize the generation of infinity values when the number of views is 0. Wilcoxon rank sum tests were used to compare the TikTok user engagement on a log scale between different feature categories. A linear mixed effects model was used to identify significant features associated with TikTok user engagement. The purposeful variable selection method was used to select important features for the final model, and Tukey’s method was used to control the familywise error rate for pairwise comparisons. Statistical analysis software R 4.3.1 (R Core Team, 2007) was used for the data analysis, with a significance level of 5% for all the analyses.

## Results

### Feature Extraction From TikTok Videos Using Large Language Models

From the 1487 e-cigarette-related videos, we randomly selected 25 TikTok videos for hand-coding video features. We compared the accuracy in extracting features between 2 popular large language models, GPT-4 and Video-LLaMA-7B, using the hand-coding video feature as the golden standard. As shown in Table S2 in Multimedia Appendix 1, for the video features we selected, the large language model GPT-4 showed much higher accuracy (83% to 100%) than the model Video-LLaMA-7B (24% to 88%). Therefore, we used GPT-4 to extract features from the remaining 1462 videos.

### User Engagement for Video Features

Using the Wilcoxon sum rank test, we compared the distribution of TikTok user engagement measures on a log scale between different categories of those identified video features (Table 1).

**Table 1.** TikTok user engagement (log-transformed) for extracted video features.

Features	Sample size, n (%)	User engagement, mean (SD)	*P* value
Promotion content			<.001
No	1264 (85.0)	4.66 (1.99)	
Yes	223 (15.0)	3.02 (2.19)	
Celebrity endorsement			.58
No	1467 (98.7)	4.41 (2.11)	
Yes	20 (1.3)	4.67 (1.93)	
Background			<.001
Car	31 (2.1)	5.48 (1.84)	
Not detectable	345 (23.2)	4.65 (2.13)	
Public space	65 (4.4)	3.06 (2.23)	
Outside	105 (7.1)	3.95 (2.11)	
Private space	827 (55.6)	4.56 (1.98)	
Shop	114 (7.6)	3.53 (2.27)	
Perceived sex			<.001
Female	483 (32.5)	4.98 (1.88)	
Male	595 (40.0)	4.42 (2.02)	
Male and female	54 (3.6)	4.47 (2.17)	
No people	355 (23.9)	3.63 (2.28)	
Social event			<.001
No	590 (39.7)	4.13 (2.20)	
Yes	897 (60.3)	4.60 (2.01)	
Young people			<.001
No	534 (35.9)	3.92 (2.16)	
Yes	953 (64.1)	4.69 (2.02)	
Lifestyle			.002
Casual	365 (24.5)	4.70 (2.01)	
Leisure	135 (9.1)	4.03 (1.89)	
No lifestyle	987 (66.4)	4.36 (2.15)	
E-cigarette device			<.001
No	905 (60.9)	4.72 (1.98)	
Yes	582 (39.1)	3.93 (2.20)	
Smoking or vaping			.04
No	1140 (76.7)	4.35 (2.09)	
Yes	347 (23.3)	4.61 (2.13)	
Talking			<.001
No	644 (43.3)	3.80 (2.20)	
Yes	843 (56.7)	4.88 (1.90)	
Singing			.02
No	1457 (98.0)	4.39 (2.11)	
Yes	30 (2.0)	5.31 (1.61)	
Dancing			.56
No	1450 (97.5)	4.41 (2.10)	
Yes	37 (2.5)	4.61 (2.31)	
Funny or silly			<.001
No	1089 (73.2)	4.16 (2.10)	
Yes	398 (26.8)	5.09 (1.95)	
Cartoon or animation			.63
No	1246 (83.8)	4.40 (2.10)	
Yes	241 (16.2)	4.47 (2.10)	
Vape trick			.05
No	1444 (97.1)	4.39 (2.10)	
Yes	43 (2.9)	5.03 (2.22)	
Contains emoji			<.001
No	1138 (76.5)	4.26 (2.15)	
Yes	349 (23.5)	4.90 (1.86)	

As shown in Table 1, TikTok videos with a background in a car versus other background categories (5.48 for car vs 3.06‐4.65 for other background categories), featuring females versus males or females and males or no people (4.98 for females vs 3.63‐4.47 for males or females and males or no people), advertising social events (4.60 vs 4.13), including young adults (4.69 vs 3.92), showing casual lifestyles (4.70 vs 4.03-4.36), without the presence of physical e-cigarette devices (4.72 vs 3.93), having the action of people smoking or vaping (4.61 vs 4.35), including people talking (4.88 vs 3.80) or singing (5.31 vs 4.39), have significantly higher user engagement than TikTok videos without those features (all *P*<.05). TikTok videos without promotion content had substantially higher user engagement than TikTok videos with promotion content (4.66 vs 3.02). In addition, TikTok videos that are funny or silly (5.09 vs 4.16) or contain emojis (4.90 vs 4.26) had significantly higher user engagement than other TikTok videos (*P*<.001). TikTok videos with features like celebrity endorsement, people dancing, cartoons or animations, and vape tricks were not significantly different from TikTok videos without those features.

### TikTok Video Features Associated With High Social Media User Engagement

**Figure 1.**
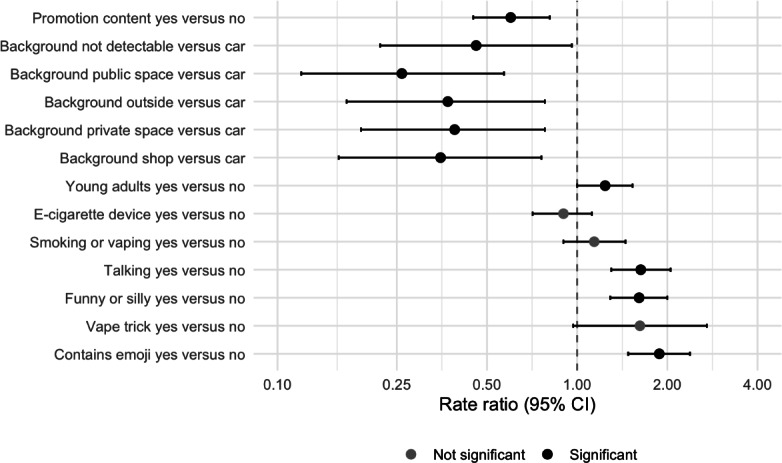
Forest plot of linear mixed effects model results with estimated rate ratio and their 95% CI for video features using the TikTok video user engagement measure on a log scale as the outcome.

In the multivariate linear mixed effects model, several video features, such as lifestyle, dancing, and singing, were not significantly associated with TikTok user engagement. According to the purposeful model selection method, these were not included in the final linear mixed effects model. Results from the linear mixed effects showed significant features associated with the TikTok user engagement after adjusting for other variables in the model (Figure 1). As the exponential of the parameter estimates were the rate ratios (RR) from the linear mixed effects model, an estimated RR of 1 or higher was classified as high engagement, and an estimated RR smaller than 1 was classified as low engagement. TikTok videos featuring young people had significantly higher user engagement than those without (estimated RR=1.24, 95% CI 1.00‐1.53; *P*=.048), corresponding to 24% greater engagement. TikTok videos with people talking had significantly higher user engagement (RR=1.63, 95% CI 1.30‐2.05; *P*<.001), meaning user engagement is 1.63 times that of videos without people talking. TikTok videos that are funny or silly had significantly higher user engagement than TikTok videos that are not funny or silly (RR=1.61, 95% CI 1.29‐2.00; *P*<.001). TikTok videos that contain emojis had significantly higher user engagement than TikTok videos without emojis (RR=1.88, 95% CI: 1.48‐2.38; *P*<.001). There is a marginally significant association between TikTok videos with vaping tricks and TikTok user engagement (RR=1.62, 95% CI 0.97‐2.71; *P*=.07). On the contrary, TikTok videos containing promotional content had significantly lower user engagement than TikTok videos without promotional content (RR=0.60, 95% CI 0.45‐0.81; *P*=.001).

**Figure 2.**
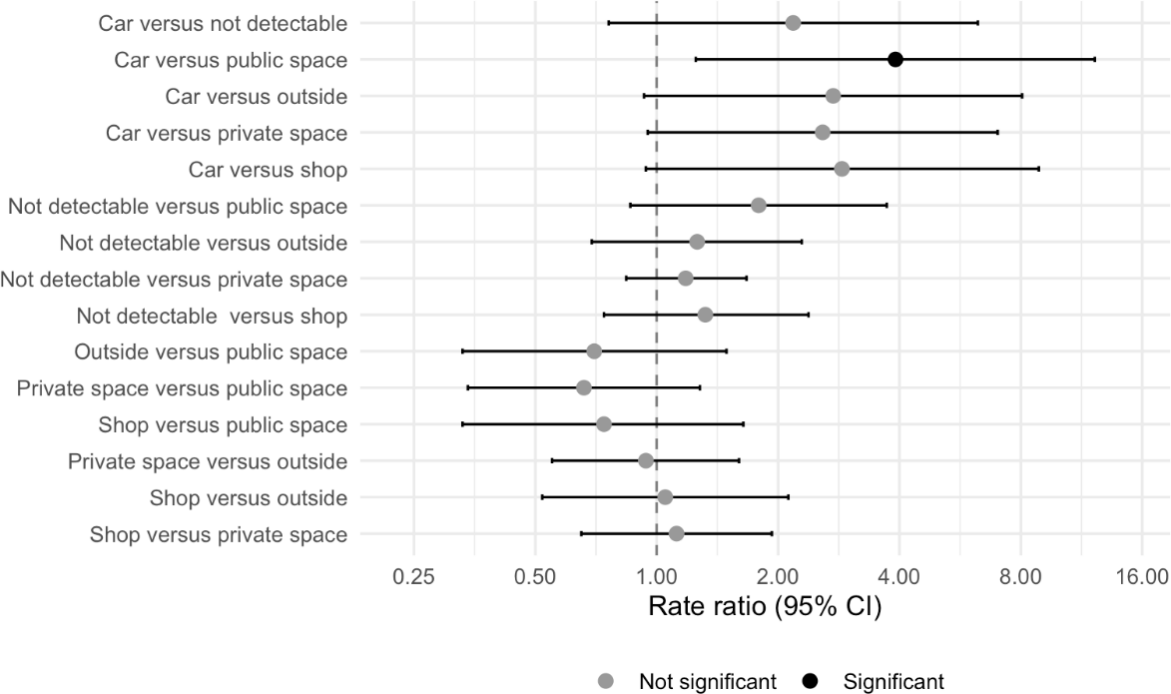
Forest plot of pairwise comparison for video background with estimated rate ratio and their 95% CI using the TikTok video user engagement measure on a log scale as the outcome.

The background setting showed a significant association with engagement. Accordingly, we estimated all pairwise contrasts between video backgrounds using Tukey’s method. Pairwise comparisons showed significant differences in TikTok user engagement among different video backgrounds (Figure 2). TikTok videos with a background in the car had significantly higher user engagement than TikTok videos with a public space background (RR=3.91, 95% CI 1.25‐12.20; *P*=.009). Other video background comparisons showed no significant differences, as their 95% CIs included 1.

## Discussions

### Principal Findings

Our study is the first to investigate video features in e-cigarette-related TikTok videos associated with high TikTok user engagement. Using artificial intelligence (large language models such as GPT-4) and generalized linear models, we extracted and identified e-cigarette-related TikTok video features associated with high user engagement, such as background settings, young adult presence, funny or silly content, and emojis. Some of these identified features (such as background setting, humorous content, and proper emojis) could be used to design future e-cigarette prevention and cessation videos to increase user engagement.

In this study, as a proof of concept, we have tried to extract a set of features from e-cigarette-related TikTok videos. Some are general video features like the background, young people, and fun. In contrast, others are more specific to e-cigarette-related videos, such as smoking or vaping and vaping tricks. While Video-LLaMA is designed to analyze videos, our results showed that GPT-4 performed better than Video-LLaMA in identifying these video features. One potential reason for this is that GPT-4 is a multimodal model trained on a much more extensive training dataset. In addition, while Video-LLaMA might be more potent in understanding video motion and temporal dynamics, GPT-4 might perform better in semantic feature extraction at the frame level, especially from still frames in videos, by leveraging cross-modal information.

The pairwise comparison results within the linear mixed effects model framework showed the differences of various video background settings in their associations with TikTok user engagement. The reduced number of significant findings for the car background in the pairwise comparisons resulted from the stricter Type I error control applied via Tukey’s method. TikTok videos with backgrounds in a car had significantly higher user engagement than TikTok videos with backgrounds in public spaces. These results showed TikTok users prefer background settings in a more closed or private space than public spaces for e-cigarette-related TikTok videos. The reason for this might be related to the vaping ban in public places, including all bars and restaurants, which was recommended by the World Health Organization (WHO) in August 2016 and implemented in most United States states, including Alabama, California, and New York [44]. The widely adopted vaping ban in public places in the United States might lead people to view vaping in public spaces, such as bars, restaurants, or medical buildings, as inappropriate. Thus, e-cigarette-related TikTok videos in public places had less user engagement than TikTok videos with car backgrounds.

E-cigarette-related TikTok videos with young adults had higher user engagement. This might be due to the significant population of e-cigarette users being youth and young adults, and most of the TikTok users being young people. However, TikTok videos that showed only the physical e-cigarette device were less attractive to users—likely because they lacked human cues, offered little narrative value, and looked like advertisements. TikTok videos that are funny or silly attract more attention. Humor seems to be an effective tool to connect with audiences on TikTok by capturing users’ interest, encouraging sharing, and fostering a sense of community. Humor could also help increase the loyalty of followers [45]. E-cigarette-related TikTok videos featuring vaping tricks appear very impressive to viewers [37], sparking curiosity among many users about how those tricks are performed [31]. Our study found a positive association between vaping tricks and user engagement that was marginally significant. While vaping tricks are commonly used to promote vaping on social media, they should be appropriately regulated or controlled to reduce their influence on susceptible social media users. Emoji is another powerful tool that attracts TikTok users’ attention. In TikTok videos, emojis could serve as visual cues to express feelings, reactions, and attitudes to effectively communicate with viewers on an emotional level and attract their engagement [46,47].

Current policy is insufficient in preventing the spread of vaping promotion videos on TikTok [23]. Identifying video features linked to higher engagement in e-cigarette–related TikTok posts has clear implications for policy and practice. For public health communication, prevention content can ethically adopt engaging formats to improve reach—eg, featuring young adults, using light emoji overlays and humorous content, and filming in relatable settings such as cars—while avoiding elements that glamorize product use.

### Limitations

Our current study has several limitations. First, the e-cigarette-related TikTok video features included in this study are only a subset of video features. Other video features, such as the audio features, should be further investigated in future studies. Our study is the first one to investigate e-cigarette-related TikTok video features associated with social media user engagement. Here, we only tested some features that could be identified using current artificial intelligence techniques; for example, the current GPT model could not detect the background settings from some of the TikTok videos. With the rapid development of artificial intelligence techniques, we believe more features could be accurately labeled by the GPT models or other more advanced models, which can be included in our future studies. Second, our list of TikTok video features may not be applicable to other social media platforms. Therefore, it is crucial for future studies to explore important features on different social media platforms, such as Instagram and YouTube. Third, the accuracy of some feature labeling by the current GPT models has not reached 100% yet. There might be mislabeling of some features in our data, which might lead to some bias in our results. The accuracy of video features could be further improved with the availability of more advanced large language or video models. In addition, because sex was inferred from perceived physical characteristics and presentation in the videos rather than self-reported identity, the measure reflects perceived sex and is subject to misclassification bias, with potential exclusion or misrepresentation of transgender, non-binary, and gender-diverse individuals. Fourth, due to the unavailability of data, we cannot determine the demographics of TikTok users who were engaged with these e-cigarette-related videos, which should be investigated using other approaches. Moreover, we do not have the information about account-level factors—such as the number of unique accounts reacting, whether an account is personal versus sponsored or brand, and whether a post was boosted or advertised—that may also influence engagement. Fifth, not all features identified in this study might be appropriate for vaping prevention videos, which should be further tested and validated in future studies. Sixth, the data collection was conducted in February 2024 and included TikTok videos posted from January 2023 to January 2024. The numbers of views, likes, shares, and comments may change over time. Although the moderate-to-strong correlations across views, likes, shares, and comments imply that the engagement ratio (likes+shares+comments)/views should remain broadly consistent, potential bias could be introduced with the changes in the engagement metrics over time. Seventh, because the TikTok API limits monthly retrievals, our 1-year sample included 1487 videos. Larger samples in future work would increase power to detect additional features associated with user engagement. Eighth, 2 coders annotated video features side-by-side rather than independently; consequently, we could not quantify inter-rater reliability (eg, Cohen Kappa). This consensus approach may inflate agreement and introduce shared-observer bias. Finally, with the rapid changes in e-cigarette marketing and social media, it is necessary to closely monitor and update new developments of social media posts with high user engagement.

### Conclusions

In this study, we showed that specific TikTok video features, including background settings (eg, car), talking, funny or silly, and use of emojis, were significantly associated with higher TikTok user engagement. These findings offer practical guidance for designing more engaging vaping-prevention videos for a broader reach of social media users.

## Supplementary material

10.2196/76265 Multimedia Appendix 1 Supplemental tables and figures, including prompts used to extract video features, accuracy comparison of the large language models, and Spearman correlations among likes, shares, comments, and views in a log scale of TikTok videos.

## Abbreviations

API: application programming interface
e-cigarette: electronic cigarette
FDA: US Food and Drug Administration
NYTS: National Youth Tobacco Survey
RR: rate ratio
RSRB: Research Subjects Review Board
STROBE: Strengthening and Reporting of Observational Studies in Epidemiology
Video-LLaMA-7B: Video Large Language Model Meta AI
WHO: World Health Organization
