# Supplementary material for: Leveraging Large Language Models to Identify Engagement-Driving Features in Vaping-Related TikTok Videos: Cross-Sectional Study
Source: J Med Internet Res. 2025 Nov 20;27:e76265. doi: 10.2196/76265 (PMC12634013; doi:10.2196/76265)
Supplement: Multimedia Appendix 1 [file jmir-v27-e76265-s001.docx]

**Supplemental Tables and Figure**

**Table S1**. Prompts used to extract video features from TikTok videos.

| **Features** | **Prompts** |
| --- | --- |
| Promotion content | Do the images describe anything related to incentives or promotion content? Answer it in one word: Yes or No. |
| Celebrity endorsement | Are these images related to any celebrity endorsement? Answer it in one word: Yes or No. |
| Background | Describe the place of the entire image in a few words. |
| Gender | Tell the gender of the person or persons involved in the images. If no person is present, say No. The answer should be in - male, female, no. |
| Social event | How many persons are present in the entire image? Answer the number alone; if there are no persons, say No. |
| Young adults | Are there any young people involved in the images? Answer it in one word: Yes or No. |
| Lifestyle | Describe the lifestyle shown in the images, such as the inclusion of friendly hotels, food, beverages, fancy cars, ...etc. |
| E-cigarette device | Are there any e-cigarette products or brands in the images? Answer it in one word: Yes or No. |
| Smoking or vaping | Is there any smoking or vaping activity involved in the images? Answer it in one word: Yes or No. |
| Talking | In the entire image, is anyone talking? Answer it in one word: Yes or No. |
| Singing | In the entire image, is anyone singing? Answer it in one word: Yes or No. |
| Dancing | In the entire image, is anyone dancing? Answer it in one word: Yes or No. |
| Funny or silly | Do the images show any funny or silly things? |
| Cartoon or animation | Do the images contain any cartoon or animation things? Answer it in one word: Yes or No. |
| Vape tricks | Does the video show any vape tricks? Answer it in one word: Yes or No. |
| Emoji usage | Do the video comments include emoji? Answer it in one word: Yes or No. |

**Table S2.** Accuracy comparison of the large language model GPT-4 with Video- LLaMA-7B in extracting features from 25 TikTok videos.

| **Features** | **GPT-4** | **Video-LLaMA – 7B** |
| --- | --- | --- |
| Promotion content | 100% | 76% |
| Celebrity endorsement | 100% | 68% |
| background | 100% | 88% |
| Gender | 96% | 80% |
| Social Event | 96% | 24% |
| Young adults | 83% | 56% |
| Lifestyle | 92% | 76% |
| E-cigarette | 100% | 68% |
| Smoking or Vaping | 96% | 76% |
| Talking | 83% | 64% |
| Singing | 100% | 44% |
| Dancing | 100% | 36% |
| Funny or Silly | 92% | 40% |
| Cartoon or Animation | 96% | 80% |
| Vape trick | 76% | 56% |
| Containing emoji | 96% | 40% |


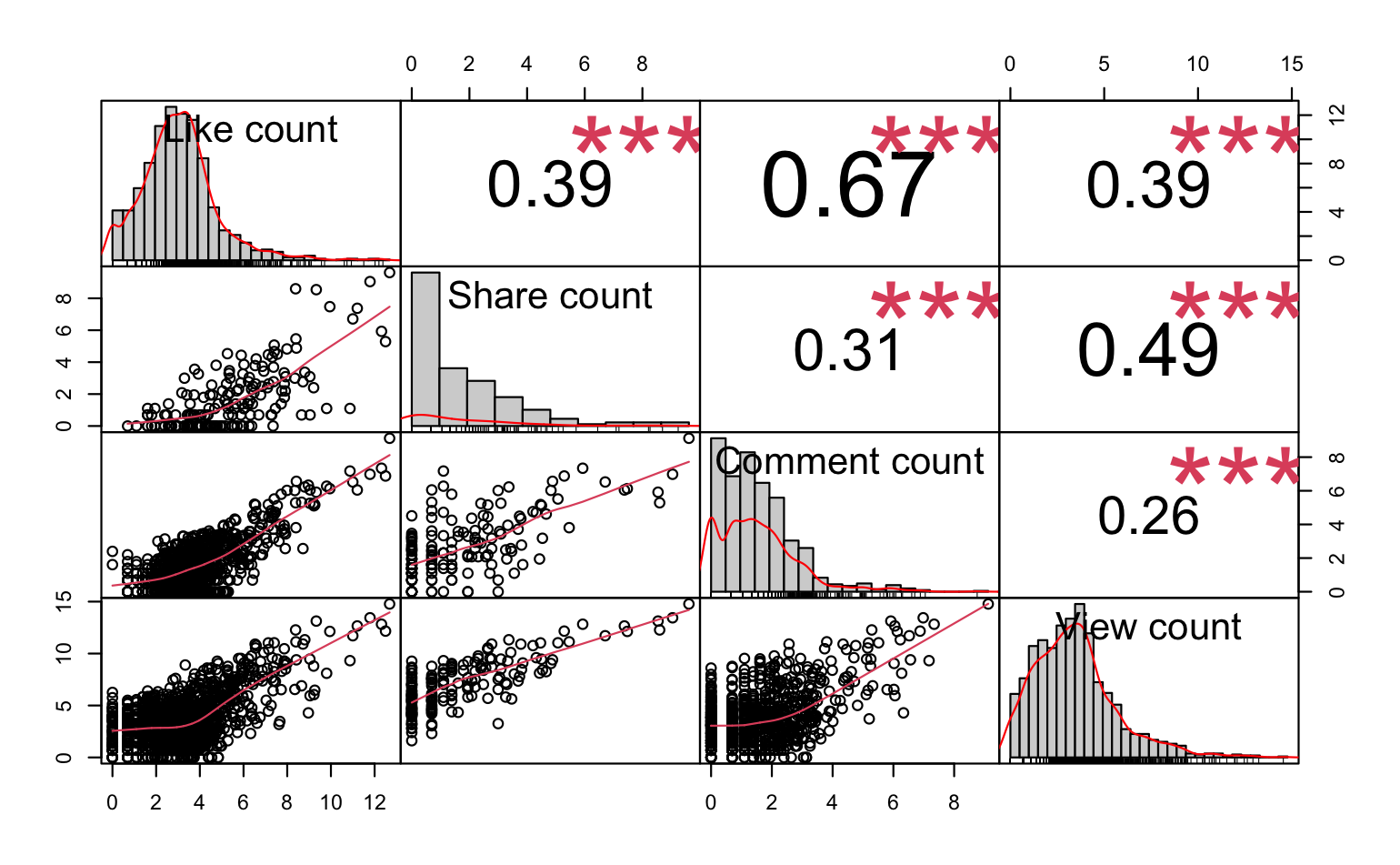


**Figure S1.** Spearman correlations among likes, shares, comments, and views in a log scale of TikTok videos. *** denotes *P*<.0001.
